# A Case of Nerve Root Radiofrequency Thermocoagulation for Pain Due to Pleural Metastasis of Lung Cancer Leading to Improvement in the Patient's Quality of Life

**DOI:** 10.1089/pmr.2023.0036

**Published:** 2023-10-18

**Authors:** Yumi Tsuzuki, Takahisa Nishiyama, Yusuke Ishida, Ryoji Maeda, Mikiko Tomino, Kiyoshige Ohseto

**Affiliations:** ^1^ Department of Anesthesiology, Tokyo Medical University, Shinjuku-ku, Japan.; ^2^ Department of Anesthesiology, Tokyo Medical University Hachioji Medical Center, Hachioji, Japan.; ^3^ Department of Anesthesiology, Showa University Hospital, Shinagawa-ku, Japan.

**Keywords:** cancer-related pain, cancer survivor, lung cancer, nerve root block, radiofrequency thermocoagulation

## Abstract

Advances in medicine have made long-term survival of cancer patients possible. Hence, it is now necessary to consider how to approach common symptoms, such as cancer-related pain, in these patients. In this study, we describe a lung cancer patient in whom relief of intractable thoracic pain caused by pleural metastasis was achieved through thoracic radiofrequency thermocoagulation (RF), improving his quality of life (QOL). The patient was a man in his 70s with right upper lobe lung cancer, left 9th −11th rib metastasis, and left thoracic pain associated with parietal pleural metastasis. The patient experienced insomnia and weight loss due to poor appetite caused by opioid analgesics and inadequate pain control. Therefore, RF was performed as interventional treatment, resulting in a decrease in the numerical rating scale score from 10/10 to 2/10, and an improvement in QOL. In cases wherein long-term survival is expected, a long-term treatment plan for chronic cancer-related pain, which has a tendency to become persistent, becomes necessary. RF for the nerve roots might be a viable option for pain caused by pleural metastasis in cancer survivors.

## Introduction

Cancer-related pain is a common symptom experienced by cancer patients. Inadequate pain control causes physical and psychological distress to patients, and also affects their overall quality of life (QOL). According to guidelines reported from Japan, the use of combinations of opioid and nonopioid analgesics is recommended for the management of cancer-related pain.^1^ Although there are many opportunities for using these medications for cancer pain, currently, there are many patients in whom achieving sufficient pain relief through pharmacological therapy alone is difficult, as well as patients who find it challenging to increase their medication or those who are forced to discontinue their use due to the side effects of opioids.

However, advances in chemotherapy and radiation therapy have made long-term survival possible compared with the past, necessitating development of a long-term treatment plan for chronic cancer-related pain. Reportedly, interventional therapy, such as nerve blocks, can be used to complement pharmacological therapy, resulting in effective pain relief while reducing the overall medication dose.^2^ In this study, we report our experience of successful achievement of good pain control through thoracic radiofrequency thermocoagulation (RF) for intractable thoracic pain caused by pleural metastasis in a lung cancer patient with expected long-term survival. We obtained written informed consent from the patient to publish this case report.

## Case Description

**FIG. 1.**
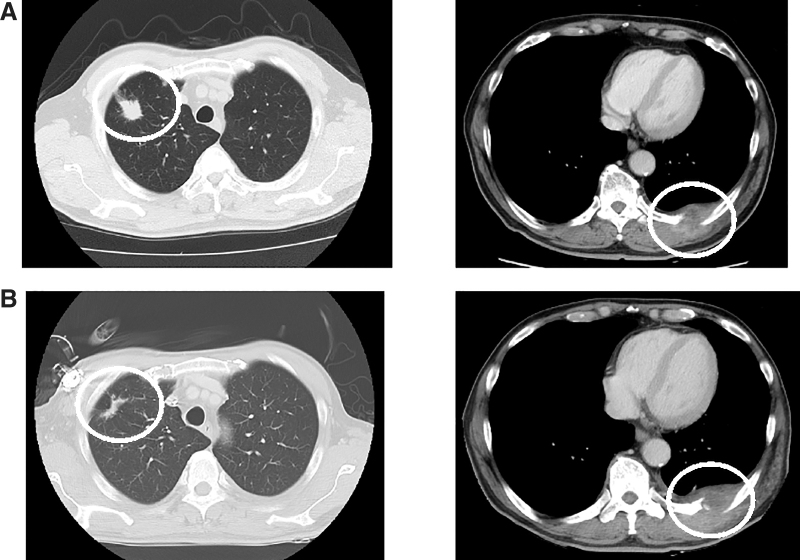
Chest computed tomography. **(A)** Tumors are found in the right lung field and left chest wall. **(B)** The tumor in the left chest wall is slightly enlarged. The red circles indicate the locations of the tumors.

A 71-year-old man was diagnosed with right upper lung lobe cancer (Fig. 1A). The patient visited our pain clinic due to left thoracic pain caused by left pleural infiltration associated with metastasis to the 9th −11th ribs. At the initial visit, the patient was taking 12 mg per day of hydromorphone hydrochloride (Narusus^®^), along with additional 2 mg hydromorphone hydrochloride (Narurapid^®^) doses as rescue medication, up to a maximum of three doses per day. However, his numerical rating scale (NRS) score indicated poor pain control, ranging from 9 to 10 on a scale of 10, and the patient also experienced insomnia and pain during movement.

**FIG. 2.**
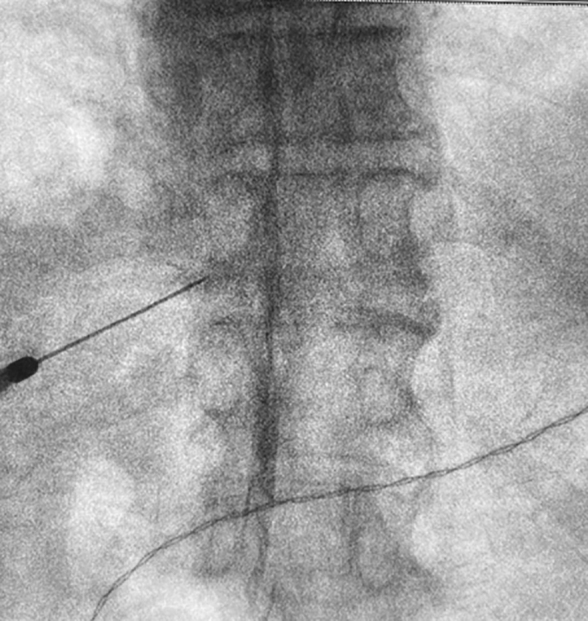
X-ray image of the thoracic spine. Radiofrequency thermocoagulation is performed under fluoroscopic guidance.

Furthermore, the patient experienced significant loss of appetite due to severe nausea, and became bedridden. Therefore, a plan was made to perform RF of the 9th −11th thoracic spinal nerve roots to achieve pain relief and reduce the medication dosage. Under fluoroscopic guidance, needles (22G, Active tip 4 mm; Hakko Medical Co., Tokyo, Japan) were inserted near the 9th −11th thoracic spinal nerve roots, and electrical stimulation (50 Hz, 0.3–0.7 V) was applied to each nerve root to reproduce the pain (Fig. 2).

Once reproduction of the pain was confirmed, 1 mL of 2% lidocaine was injected. After confirming the extent of pain improvement, RF was performed for 90 seconds at a set temperature of 80°C. We ensured that there were no neurological abnormalities, such as paralysis in the lower extremities, while performing the procedure. The patient confirmed pain relief during the RF procedure, and his vital signs remained stable. The procedure was completed in ∼20 minutes. There were no postoperative complications, such as severe sensory or perception disorders.

NRS scores improved from 10/10 to 5/10 after the procedure. One week later, an additional RF procedure was performed at the other sites causing pain, resulting in further improvement to an NRS score of 2/10. Pain during movement was also reduced. The use of rescue medication was discontinued after the two RF procedures. Nausea was relieved and QOL was improved after RF was performed. After the treatments, an improvement in activities of daily living (ADL) was observed to the extent that the patient could walk. He subsequently underwent several chemotherapy sessions as an outpatient.

Palliative radiotherapy was also used. Six months later, computed tomography (CT) scan revealed reduction in the size of the tumor in the right upper lobe, but with a mild increase in the left pleural tumor (Fig. 1B). However, both rest pain and movement-related pain were controlled, with an NRS score of 2/10, and there was no worsening of pain. Subsequently, two months later, the patient experienced recurrence of the chest pain. His NRS score was high, ranging from 8 to 10 on a scale of 10, and the pain was localized to the same area as during the initial visit.

Chest CT scan showed tumor progression and rib destruction, indicating pain derived from bone destruction and diminished effect of the previous RF treatment. Therefore, a repeat RF procedure was performed for the 9th −11th thoracic spinal nerve roots. After RF, his NRS score decreased to 3–4/10, allowing adjustment of the opioid dose in home health care.

## Discussion

Cancer remains the leading cause of death in Japan, although the age-adjusted mortality rate has decreased due to advances in cancer treatment, leading to an increase in the number of cancer survivors.^3^ Even among cases with distant metastases, which were previously associated with short survival, there is now an increasing number of patients who live for an extended period of time. As a result, there is a growing need to actively pursue methods to improve ADL and QOL. Opioids can be administered without restrictions in the management of cancer pain.

However, the extended life span of these patients is sometimes marred by inadequate pain control. Furthermore, pain caused by tumor infiltration into the chest wall or pleura often becomes difficult to manage and might worsen over time.^4^ In the present case, despite opioid medication for controlling the pain resulting from pleural metastasis, achieving effective pain control was challenging. When managing cancer pain, interventional therapies offer potential benefits in cases in whom the effectiveness of opioid analgesics is limited.^5^

Oh et al. reported that RF targeting the nerve roots for chest wall pain might provide pain relief for several months.^6^ In the present case, despite an increase in the size of the chest wall tumor over a six-month period, the patient's pain was controlled, with NRS scores of 2–4/10, and with maintenance of ADL and QOL while receiving outpatient chemotherapy. Moreover, he did not experience opioid-related side effects, such as nausea or drowsiness. Since the pain was relieved for approximately seven months after the period of RF treatment, the RF procedure was considered effective.

RF works on the principle of coagulating and destroying nerve fibers that transmit pain using heat generated by high-frequency electrical currents.^7,8^ One advantage of RF is that it allows the reproduction and confirmation of pain through electrical stimulation.^9^ Moreover, RF can selectively damage nerves locally in the vicinity of the needle tip, the effects of which become apparent immediately after the procedure. Another advantage is that RF can be performed multiple times at the same site or at other higher level nerve roots.

However, caution is required during RF procedures due to the risks of sensory and motor impairment, as well as the possibility of subarachnoid puncture. Another alternative interventional therapeutic procedure is pulsed radiofrequency therapy.^10,11^ Since the number of cancer survivors is expected to increase in the future, there is a need to develop pain management strategies for these patients. In addition to pharmacological treatments, interventional therapies, such as nerve root RF, have the potential to maintain a high level of ADL and QOL without affecting the patient's lifespan.

The treatment of cancer-related pain often requires a combination of various therapies tailored to the individual's pain. In the present case, RF performed for cancer-related pain achieved favorable analgesic effects.

## Abbreviations Used

ADL: activities of daily living
CT: computed tomography
NRS: numerical rating scale
QOL: quality of life
RF: radiofrequency thermocoagulation
